# Epigallocatechin-3 gallate prevents pressure overload-induced heart failure by up-regulating SERCA2a via histone acetylation modification in mice

**DOI:** 10.1371/journal.pone.0205123

**Published:** 2018-10-04

**Authors:** Lifei Liu, Weian Zhao, Jianxia Liu, Yi Gan, Lingjuan Liu, Jie Tian

**Affiliations:** 1 Department of Anesthesiology, Children’s Hospital of Chongqing Medical University, Chongqing, PR China; 2 Department of Cardiology, Heart Center, Children's Hospital of Chongqing Medical University, Chongqing, PR China; 3 Ministry of Education Key Laboratory of Child Development and Disorders, PR China; 4 Key Laboratory of Pediatrics in Chongqing, Chongqing, PR China; 5 Chongqing International Science and Technology Cooperation Center for Child Development and Disorders, Chongqing, PR China; Cleveland Clinic, UNITED STATES

## Abstract

Heart failure is a common, costly, and potentially fatal condition. The cardiac sarcoplasmic reticulum Ca-ATPase (SERCA2a) plays a critical role in the regulation of cardiac function. Previously, low SERCA2a expression was revealed in mice with heart failure. Epigallocatechin-3-gallate (EGCG) can function as an epigenetic regulator and has been reported to enhance cardiac function. However, the underlying epigenetic regulatory mechanism is still unclear. In this study, we investigated whether EGCG can up-regulate SERCA2a via histone acetylation and play role in preventing heart failure. For this, we generated a mouse model of heart failure by performing a minimally invasive transverse aortic constriction (TAC) operation and used this to test the effects of EGCG. The TAC+EGCG group showed nearly normal cardiac function compared to that in the SHAM group. The expression of SERCA2a was decreased at both the mRNA and protein levels in the TAC group but was enhanced in the TAC+EGCG group. Levels of AcH3 and AcH3K9 were determined to decrease near the promoter region of *Atp2a2* (the gene encoding SERCA-2a) in the TAC group, but were elevated in the TAC+EGCG group. Meanwhile, HDAC1 activity and binding near the *Atp2a2* promoter were increased in the TAC group but decreased with EGCG addition. Further, binding levels of GATA4 and Mef2c near the *Atp2a2* promoter region were reduced in TAC hearts, which might have been caused by histone hypoacetylation; this was reversed by EGCG. Together, upregulation of SERCA2a via the modification of histone acetylation plays a role in EGCG-mediated prevention of pressure overload-induced heart failure, and this might represent a novel pharmacological target for the treatment of heart failure.

## 1. Introduction

Heart failure is defined as “a clinical syndrome characterized by systemic perfusion which is inadequate to meet the body’s metabolic demands as a result of impaired cardiac function”. This remains an increasing global disease, with an estimated prevalence of > 37.7 million individuals globally [1]. Although substantial efficacy is associated with evidence-based therapies such as angiotensin-converting enzyme inhibitors (ACEIs), angiotensin receptor blockers (ARBs), aldosterone antagonists, and β-adrenergic receptors blockers (β-blockers), poor clinical outcomes remain a major public-health issue, and the prevalence of this syndrome continues to expand globally [2]. Thus, novel pharmacological agents aimed at new targets or based on new mechanisms are needed.

The cardiac sarcoplasmic reticulum Ca-ATPase (SERCA2a), which regulates intracellular calcium handling, plays a critical role in initiating cardiac contraction and relaxation [3]. The expression levels of SERCA2a mRNA and protein were shown to be significantly decreased in animal models of pressure overload-induced heart failure [4–6]. Furthermore, studies on human hearts have suggested that decreases in SERCA mRNA, protein, or activity are closely correlated with suppressed myocardial function and impaired force-frequency response [7–10]. As such, increasing the expression or activity of SERCA2a by genetic modification is considered a revolutionary approach to address the treatment of heart failure [11, 12]. Studies have shown that SERCA2a overexpression in myocytes via adenoviral gene transfer results in increased contractility and faster relaxation of the transient calcium [13, 14]; it can also improve myocardial systolic and diastolic function in animals with pressure overload-induced heart failure [15–17]. Recently, histone epigenetic modification was revealed to regulate SERCA2a in a mouse model of pressure overload-induced heart failure [18], indicating that the epigenetic regulation of SERCA2a might represent a new mechanism to prevent heart failure.

Epigallocatechin-3-gallate (EGCG), derived from green tea, has been shown to have multiple effects on human pathological and physiological processes, especially cardiovascular diseases [19, 20]. EGCG significantly enhances the contractility of intact murine myocytes by increasing sarcoplasmic reticulum Ca(2+) content [21]. Meanwhile, EGCG also plays a considerable role in epigenetic regulation; for example, it can inhibit class I histone deacetylases (HDACs) to increase levels of acetylated histone and inhibit DNA methyltransferase to reactivate methylation-silenced genes [22–24]. However, the epigenetic mechanism through which EGCG contributes to the prevention of heart failure remains unclear.

We hypothesized that the upregulation of SERCA2a, via EGCG-induced histone hyperacetylation, plays a role in preventing heart failure. To verify this hypothesis, we performed transverse aortic constriction (TAC) to create an animal model of heart failure. We then used this model to investigate the effects of EGCG treatment on the localization of SERCA2a, as well as other markers of epigenetic regulation, to DNA near the *SERCA2a* promoter.

## 2. Materials and methods

### 2.1. Experimental animals

Adult male mice C57BL/6 (8–10-weeks old) were purchased from the Animal Center of Chongqing Medical University (Chongqing, China). All experimental procedures involving animals were approved by the Animal Care and Use Committee at Chongqing Medical University. The mice were maintained in individually-ventilated cages (at 25°C and 55–65% humidity) with a 12-h light/dark cycle and free access to standard laboratory mouse chow. The animals were randomly divided into four groups, namely SHAM, TAC, TAC+EGCG, and SHAM +EGCG, with 8–10 mice in each. TAC+EGCG- and SHAM+EGCG-group mice were treated with a single dose (50 mg/kg/day) of EGCG (Selleck, USA) via intraperitoneal injection after surgery once per day for 12 weeks.

### 2.2. Minimally invasive transverse aortic constriction (TAC)

A minimally invasive TAC method [25] was used in our study. The mice were anesthetized via inhalation of 1.5–2.5% isoflurane, and their body temperature was maintained between 36 and 37°C throughout the procedure using a heating pad. A horizontal skin incision of 7–10 mm in length was made at the level of the suprasternal notch. Then, the thyroid was retracted and the sternum was exposed. A 5-mm longitudinal cut was made down the sternum and the thymus was retracted to allow visualization of the aortic arch under low-power magnification. A 6–0 silk suture was passed through the aortic arch between the origin of the innominate and left common carotid arteries, guided by a curved 27-gauge needle. Another 27-gauge needle was placed next to the aortic arch, and the suture was tied neatly around the needle and aorta. After ligation, the needle was removed. The skin was closed with a 4–0 nylon suture, and the mice were allowed to recover fully in a clean cage on a heating pad (S1 Text). Buprenorphine was administered subcutaneously once per day for 3 days for post-operative analgesia. The animal’s post-operative health and surgical site were observed and recorded twice per day for 7 days until the sutures was removed, then once per day for 3 months.

### 2.3. Echocardiography

Echocardiography studies were performed with a Vevo 2100 high-resolution imaging system (VisualSonics, Toronto, Canada). All measurements were performed by the same examiner. The mice were anesthetized with 1–1.5% isoflurane and placed on a heating pad to maintain body temperature at 36.5–37.5°C. Hair on the precordial region was removed with depilatory cream. B-mode images were taken to measure the ventricular and aortic structure and M-mode images were taken to measure the ventricular function. P-mode images were taken to measure the velocity of blood flow (S2 Text). All data were analyzed to evaluate the effects of TAC treatment.

The efficacy of TAC was tested by transthoracic Doppler echocardiography 3 days after the procedure. The mice were sacrificed with isoflurane and the hearts were harvested after transthoracic echocardiography was performed 12 weeks after the operation.

### 2.4. Hematoxylin and eosin (HE) staining

Heart tissues were fixed with 4% paraformaldehyde, dehydrated with alcohol, embedded in paraffin, sectioned into 5-mm slices, and then stained with hematoxylin and eosin. Photographs of the ventricular sections from the four groups of mice were taken at 400× magnification (Nikon, Tokyo, Japan)

### 2.5. Quantitative real-time PCR (qPCR)

Total RNA was extracted using the RNA extract kit (Bioteck, Beijing, China). Single-strand cDNA was reverse transcribed from 500–1,000 ng of RNA using oligo dT-adaptor primers and the AMV reverse transcriptase kit (TaKaRa, Otsu, Japan). cDNA was detected by performing a quantitative real-time polymerase chain reaction (RT-PCR) assay with the SYBR Green RealMasterMix kit (Tiangen, Beijing, China). The mRNA expression levels of *SERCA2a* were quantified and *GAPDH* was used as an endogenous ‘housekeeping’ gene to normalize RNA levels across samples. The procedures were performed in accordance with the manufacturer’s instructions. The primer sequences were 5′-TCGACCAGTCAATTCTTACAGG-3′ and 5′-CAGGGACAGGGTCAGTATGC-3′ for *SERCA2a*, and 5′-AAGAAGGTGGTGAAGCAGGCATC-3′ and 5′- CGGCATCGAAGGTGGAAGAGTG-3′ for *GAPDH*.

### 2.6. Western blot analysis

In brief, total protein was extracted from the cardiac tissue using a protein extraction kit (KeyGEN Bio-TECH, China) and then quantified using a BCA assay (BioTeke Biotechnology, China). Total protein (50 μg per lane) was separated on a 12% SDS-PAGE gel and transferred to a PDVF membrane. Proteins bound to the PDVF membrane were analyzed by western blotting using primary antibodies specific for SERCA2a (Abcam, USA) and GAPDH (Arigo, Taiwan). The band intensity was analyzed and quantified using a G-BOX imaging system (Syngene, UK).

### 2.7. Chromatin immunoprecipitation (ChIP) assay

ChIP experiments were conducted using a ChIP assay kit (ChIP Kit-one step, Abcam, USA). After the homogenization of cardiac tissues, 1% formaldehyde (SigmaAldrich, St. Louis, MO, USA) was added to the samples to cross-link proteins to DNA. The cross-linked DNA was then fragmented into small fragments (500–1000 bp) using an ultrasound (Bioruptor UCD-200). Next, the protein–DNA complexes were precipitated using monoclonal antibodies specific for acetylated histone 3 (AcH3), lysine 4 in H3 (AcH3K4), lysine 9 in H3 (AcH3K9), HDAC1, GATA4, and Mef2c (Abcam, Cambridge, USA). Anti-RNA polymerase antibodies were used as a positive control and anti-mouse IgG was used as a negative control. The total column input also served as a positive control. Next, cross-linked protein–DNA complexes were removed and the DNA was extracted. Specific quantitative real-time PCR (qPCR) primers were designed to determine levels of AcH3, AcH3K4, and AcH3K9 near the proximal promoter regions of *Atp2a2*. The qPCR primer sequences used to amplify the *SERCA2a* promoter were as follows: 5′-AGCCAAGGACACCAGTGC-3′ and 5′-GGGATAGAGCGCGGAGTT- 3′.

### 2.8. HDAC1 activity assay

HDAC1 activity was determined using a HDAC1 Activity Assay kit (BioVision, 10Mountain View, CA, USA) according to the manufacturer’s instruction. Briefly, after protein extraction and protein concentration measurement, 6 microliters of HDAC1 antibody or control antibody were added to each 500 microliter reaction, and the reaction was incubated overnight at 4°C. Protein-A/G (25 microliters) was added to each reaction prior to incubation for 1 h at 4°C. Each reaction was then centrifuged at 14,000 × *g* for 10 s at 4°C, and the supernatant was discarded. The reaction compound containing HDAC Assay Buffer and HDAC1 substrate was subsequently added to each reaction and incubated for 2 h at 37°C. Then, 20 microliters of the Developer was added to each tube, which was incubated for 30 min 37°C. The AFC (7-amino-4-trifluoromethy coumarin) Standard was diluted as described by the manufacturer’s instructions, and for 100 microliters of each reaction, the fluorescence at Ex/Em = 380/500 nm (SYNERGY/H1 micropalte reader, BioTek) was read. The AFC standard curve was plotted and the sample reading was applied to the AFC Standard Curve to obtain B pmol of AFC in the sample wells. The calculation of sample HDAC1 activity is shown as follows:

HDAC1 activity = 2 × B / TS (pmol/min/mg = mU), where B = AFC amount from the Standard Curve, T = reaction time = 120 min, and S = protein amount.

### 2.9. Data analysis

Quantitative data are shown as the means ± SE. Statistical significance associated with differences between mean values was analyzed by performing two-way or three-way ANOVA with a post hoc Bonferroni-Dunn test for multiple comparisons. *P* < 0.05 was considered significant.

## 3. Results

### 3.1. Evaluation of the TAC operation in mice

**Fig 1:**
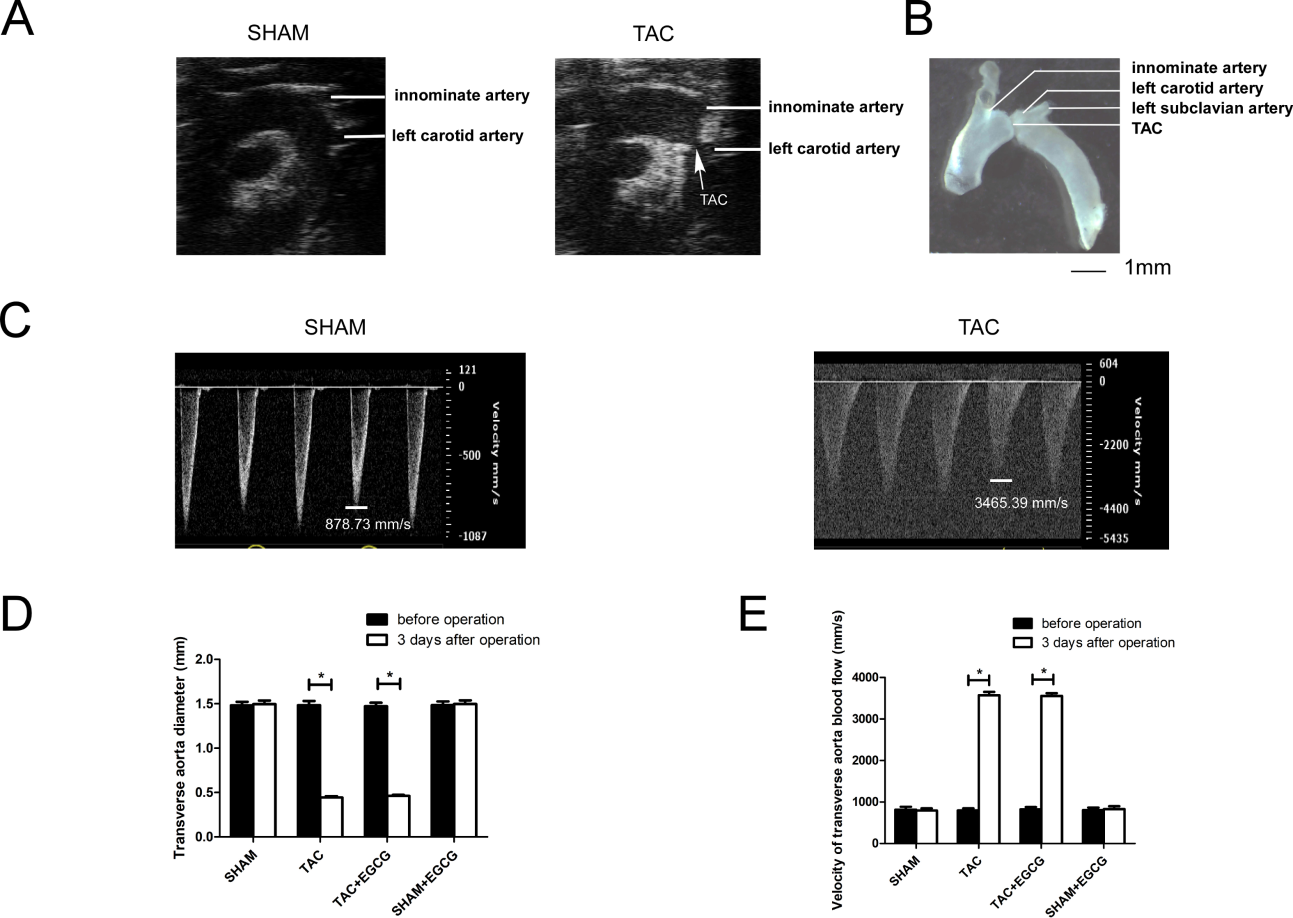
Efficacy of transverse aortic constriction (TAC) operation in mice. **A**) Representative B-mode ultrasound image of the SHAM and TAC groups. **B**) Representative anatomical image of the TAC model. **C**) Representative pulse Doppler ultrasound image of the transverse aorta blood flow in the SHAM and TAC groups. **D**) The transverse aorta diameter decreased significantly after the TAC operation with or without epigallocatechin-3-gallate (EGCG) treatment (S1 Table). **E**) The velocity of the transverse aorta blood flow (P-mode ultrasound image) increased significantly after TAC with or without EGCG (S1 Table). Values are presented as the mean ± SE. *, *P* < 0.05 by two-way ANOVA; n = 8 per group.

Echocardiograph images clearly showed reduced aortic diameter at the transverse constriction site between the innominate and left carotid arteries after TAC (Fig 1A). The constriction was further confirmed by anatomical dissection images of the aorta in TAC mice (Fig 1B). The reduced transverse aorta diameter achieved the same level after TAC between the TAC and EGCG+TAC groups (Fig 1D), resulting in the same increase in transverse aorta blood flow between these groups (Fig 1E).

### 3.2. EGCG prevents heart failure in TAC mice

**Fig 2:**
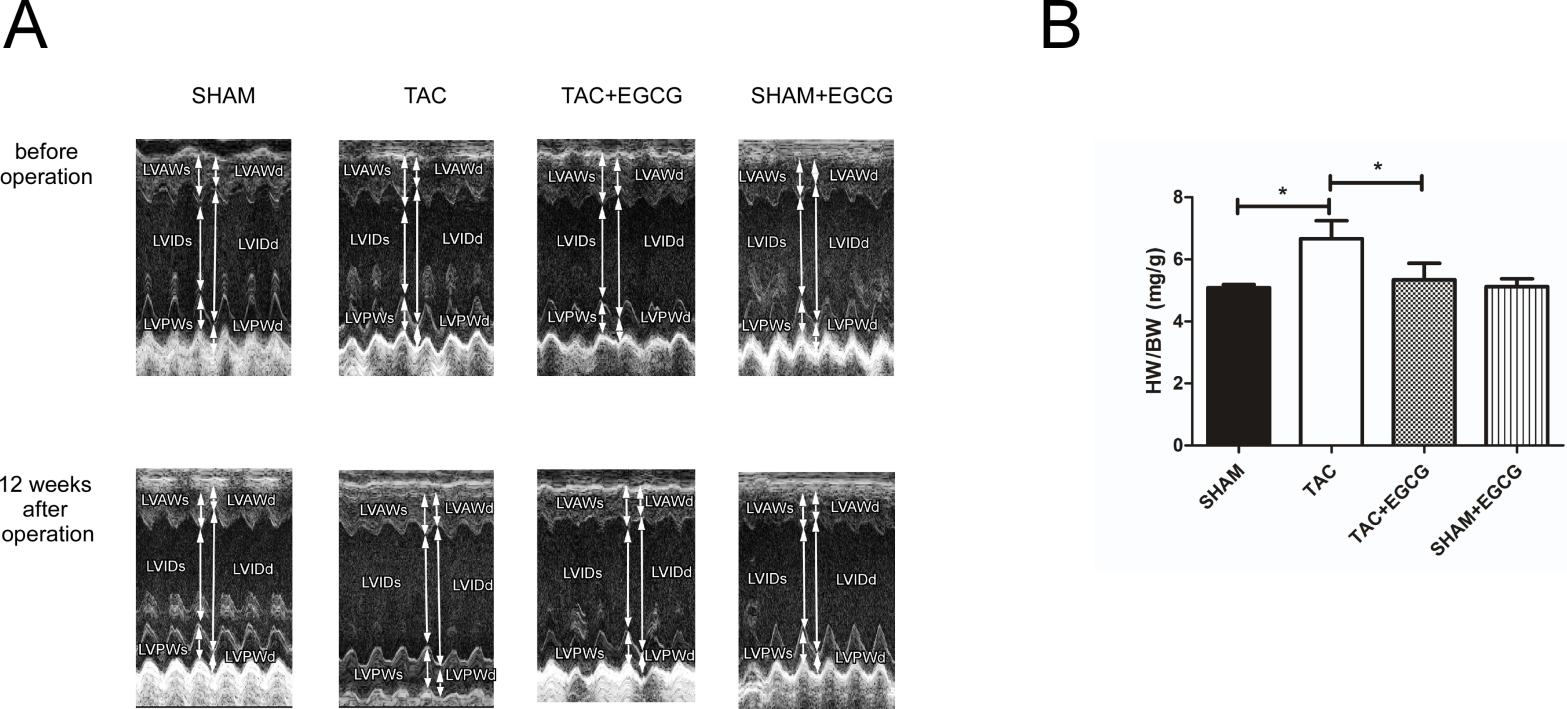
Epigallocatechin-3-gallate (EGCG) prevents heart failure induced by pressure overload. **A**) Representative M-mode ultrasound image of each group. Left ventricular anterior wall (LVAW), left ventricular internal diameter (LVID), and left ventricular posterior wall (LVPW) were increased significantly in the transverse aortic constriction (TAC) group. **B**) The heart weight to body weight ratio (HW/BW) was also higher in the TAC group than in the other groups at 12 weeks post-operation. Values are presented as the mean ± SE. *, *P* < 0.05 by two-way ANOVA; n = 8 per group.

**Fig 3:**
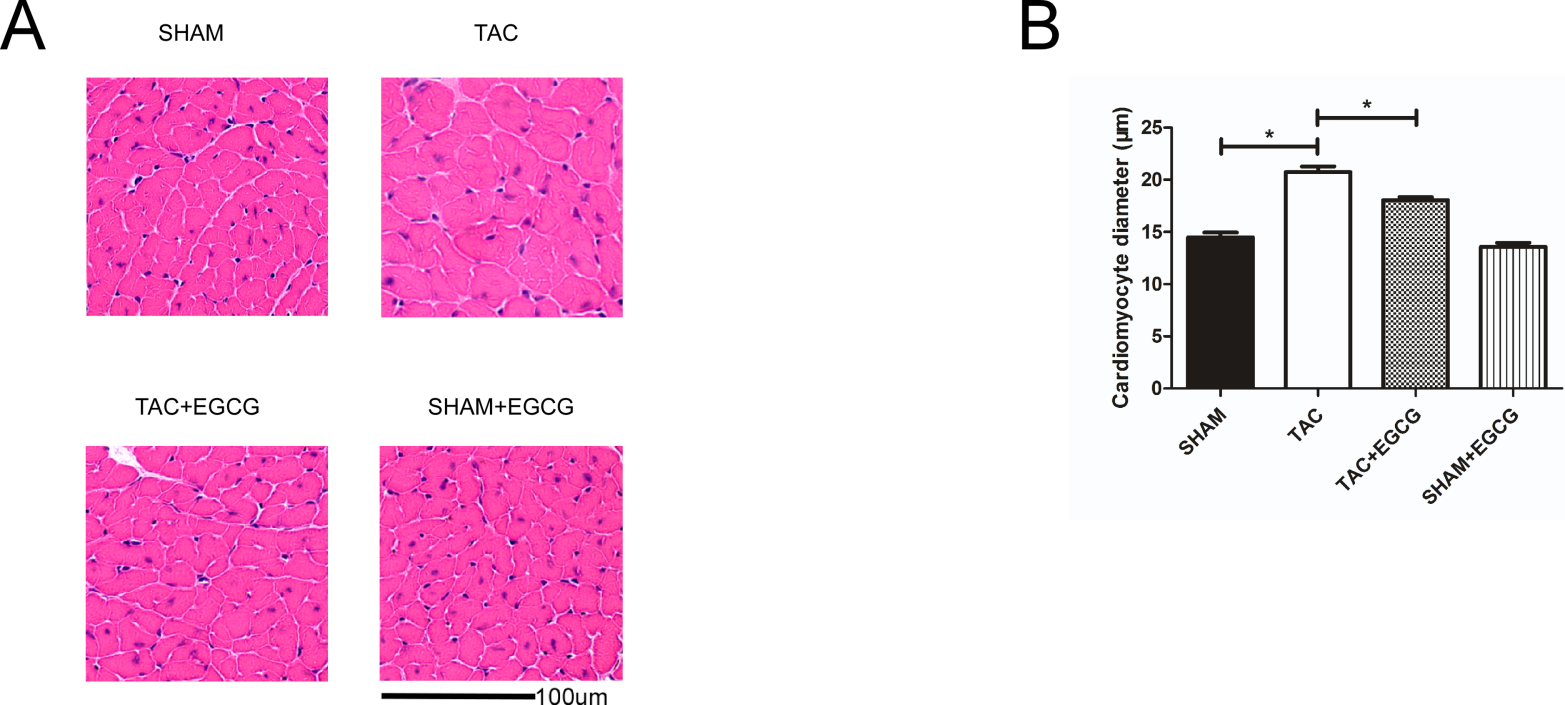
Epigallocatechin-3-gallate (EGCG) prevents myocardial hypertrophy induced by pressure overload. **A**) HE staining of left ventricle section showing that the cardiomyocyte size was larger in the transverse aortic constriction (TAC) group, and **B**) quantification of the cardiomyocyte diameter showing larger cardiomyocytes in the TAC group Values are presented as the mean ± SE. *, *P* < 0.05 by two-way ANOVA; n = 8 per group.

A non-invasive echocardiograph measurement was used to assess changes in heart wall thickness before and 12 weeks after the operation. Compared to that before the operation, the thickness of the left ventricular anterior wall (LVAW) and left ventricular posterior wall (LVPW) increased in TAC group hearts, but did not differ significantly in the other groups (Table 1, Fig 2A, S2 Table). Heart weight normalized to body weight (HW/BW) is an index to evaluate cardiac hypertrophy. Consistent with the changes in wall thickness, HW/BW increased at 12 weeks after TAC in the TAC only group, indicating a response to increased afterload; however, in the TAC+EGCG group, only a marginal insignificant increase in HW/BW was observed, indicating a compensatory effect of EGCG in response to long-term high afterload stress (Fig 2B). This was confirmed by HE staining of heart sections (Fig 3A and 3B). Before TAC, the non-invasive echocardiograph did not detect any functional differences among the groups. The left ventricular chamber was dilated, and the ejection fraction (EF) and fraction shortening (FS) were decreased in the TAC group but not in TAC+EGCG group, suggesting that EGCG preserved heart function (Table 1, Fig 2A, S2 Table).

**Table 1:** Echocardiographic parameters before and 12 weeks after operation.

Groups	SHAM (n = 8)		TAC (n = 8)		TAC+EGCG (n = 8)		SHAM+EGCG (n = 8)	
	Before operation	12 W after operation	Before operation	12 W after operation	Before operation	12 W after operation	Before operation	12 W after operation
LVAWd, mm	0.85 ± 0.02	0.91 ± 0.03	0.92 ± 0.03	1.32 ± 0.23*	0.91 ± 0.13	1.03 ± 0.06	0.90 ± 0.04	0.92 ± 0.05
LVPWd, mm	0.91 ± 0.06	0.97 ± 0.06	0.94 ± 0.05	1.31 ± 0.08*	0.93 ± 0.04	0.99 ± 0.07	0.91 ± 0.04	0.92 ± 0.04
LVIDd, mm	3.73 ± 0.08	3.76 ± 0.06	3.73 ± 0.09	4.04 ± 0.05*	3.73 ± 0.09	3.80 ± 0.13	3.74 ± 0.12	3.72 ± 0.14
EF, %	63.48 ± 1.12	63.29 ± 1.05	62.99 ± 1.81	41.37 ± 7.79*	63.01 ± 1.79	62.24 ± 3.07	62.25 ± 5.34	61.35 ± 3.73
FS, %	37.80 ± 3.15	35.71 ± 1.90	38.93 ± 1.22	21.34 ± 1.02*	38.80 ± 2.77	36.01 ± 1.71	38.25 ± 2.08	36.03 ± 1.19

VAW, left ventricular anterior wall; LVID, left ventricular internal diameter; LVPW, left ventricular posterior wall; d, diastole; LVEF, left ventricular ejection fraction; LVFS = left ventricular fraction shortening; W, weeks. LVAW, LVID, and LVPW were increased significantly in the TAC group; LVEF and LVFS were decreased significantly in the TAC group. Values are presented as the mean ± SE.

*, *P* < 0.05 compared with before and 12W after operation in the TAC group based on three-way ANOVA.

### 3.3. EGCG prevents the TAC-induced decrease in SERCA2a expression

**Fig 4:**
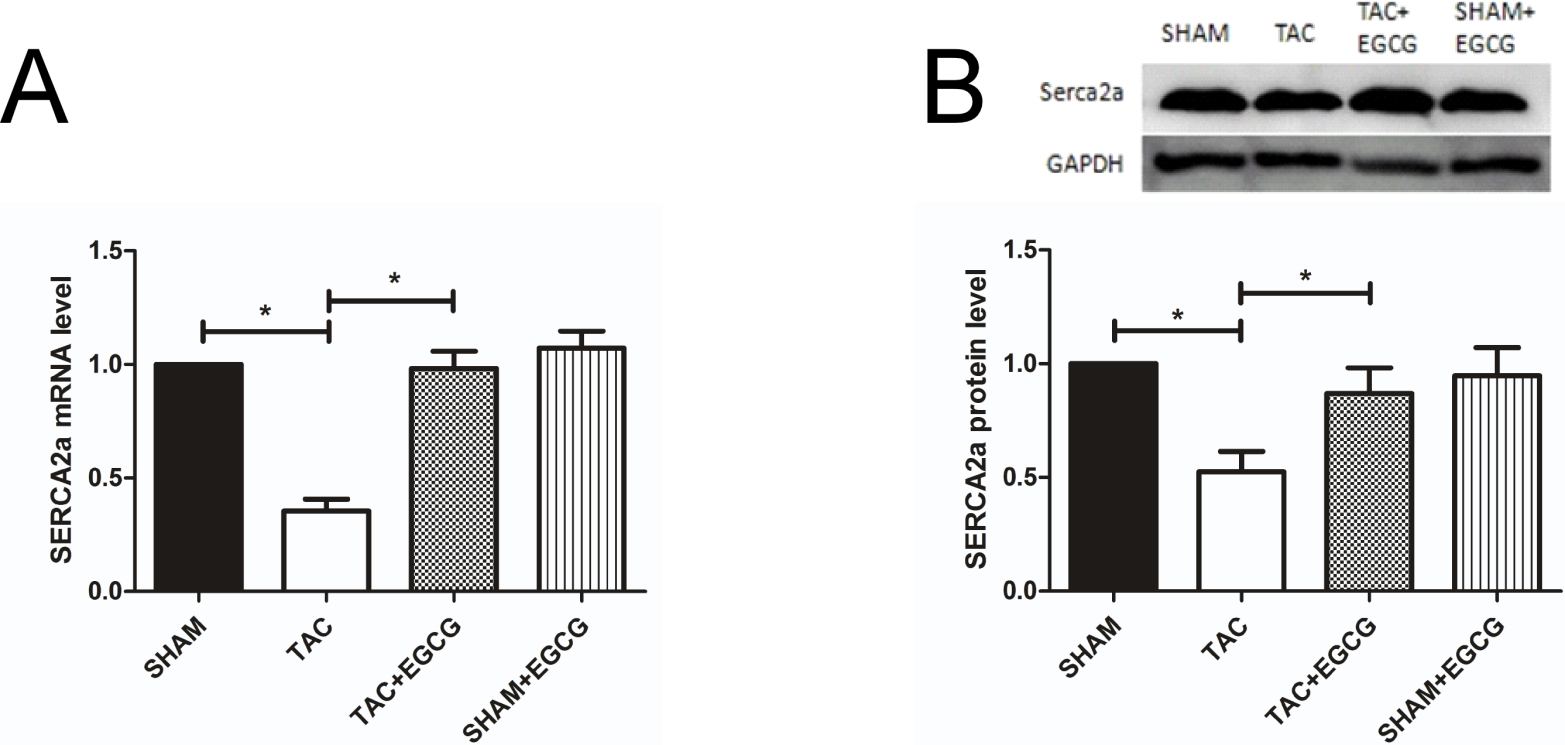
Epigallocatechin-3-gallate (EGCG) treatment increases the expression of SERCA2a upon heart failure. **A**) *SERCA2a* mRNA expression was lower in the transverse aortic constriction (TAC) group than in the other groups. **B**) SERCA2a protein expression was also lower in the TAC group than in the other groups. Values are presented as the mean ± SE. *, *P* < 0.05 by two-way ANOVA; n = 8 per group.

As shown in Fig 4A, *SERCA2a* mRNA expression was decreased in the TAC group compared to that in the SHAM group. However, this effect was almost completely reversed with EGCG treatment, as *SERCA2a* mRNA levels returned to those observed in the SHAM group. The expression of *SERCA2a* in the SHAM+EGCG group was not significantly changed as compared to that in the SHAM group. Western blot assays showed similar results (Fig 4B). These results indicate that EGCG can prevent the downregulation of SERCA2a induced by TAC.

### 3.4. Histone acetylation near the promoter region of *Atp2a2* (encoding SERCA-2a)

**Fig 5:**
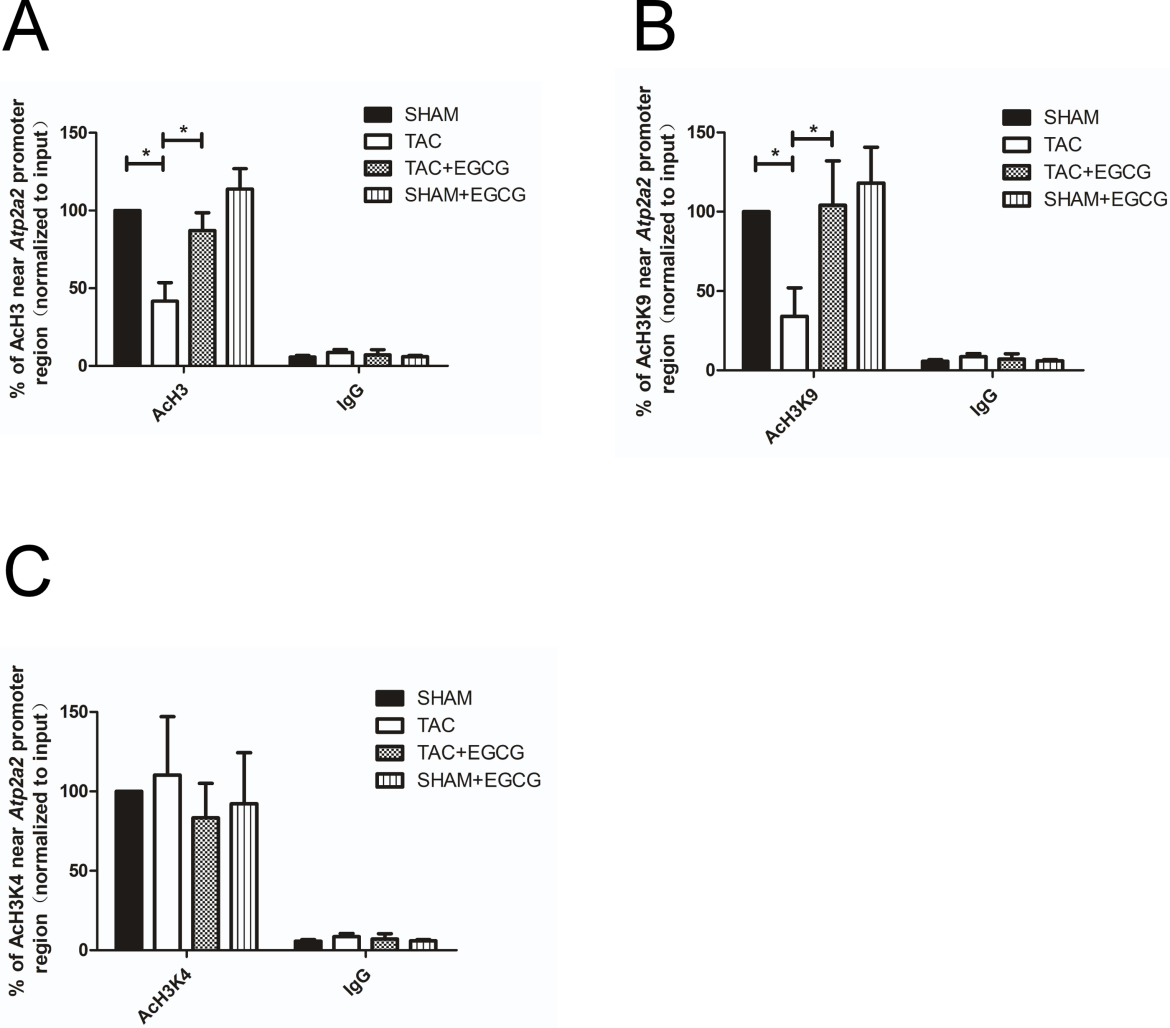
Epigallocatechin-3-gallate (EGCG) reverses the hypoacetylation of AcH3 and AcH3K9 and inhibits HDAC1 binding near the *Atp2a2* promoter region in mouse cardiac tissue after heart failure. **A**) AcH3 binding near the *Atp2a2* promoter region was significantly decreased in the transverse aortic constriction (TAC) compared to that in the other groups, as assessed by ChIP-qPCR. **B**) AcH3K9 binding near the *Atp2a2* promoter region was also significantly decreased in the TAC group compared to that in the other groups. **C**) AcH3K4 binding near the *Atp2a2* promoter region was not obviously different among the four groups. Values are presented as the mean ± SE. *, *P* < 0.05 by two-way ANOVA; n = 8 per group.

Modification of histone acetylation or deacetylation is a reversible dynamic process, and it is a crucial component of epigenetic regulation. To explore the potential mechanism underlying the reduced SERCA2a expression in TAC mice, we assessed the acetylation levels of H3, H3K4, and H3K9 near the promoter region of *Atp2a2* using ChIP-qPCR assays. As shown in Fig 5A, AcH3 levels near the promoter elements of *SERCA2a* were decreased in the TAC group compared to those in the SHAM group. In the TAC+EGCG group, levels were enhanced and reached those observed in the SHAM group. The pattern of AcH3K9 was similar to that of AcH3 among the groups, indicating that reduced SERCA2a expression was mediated by modified histone acetylation (Fig 5B). However, the levels of AcH3K4 were not significantly different among the groups (Fig 5C).

### 3.5. HDAC1 enzyme activity and binding level of HDAC1 near the *Atp2a2* promoter

**Fig 6:**
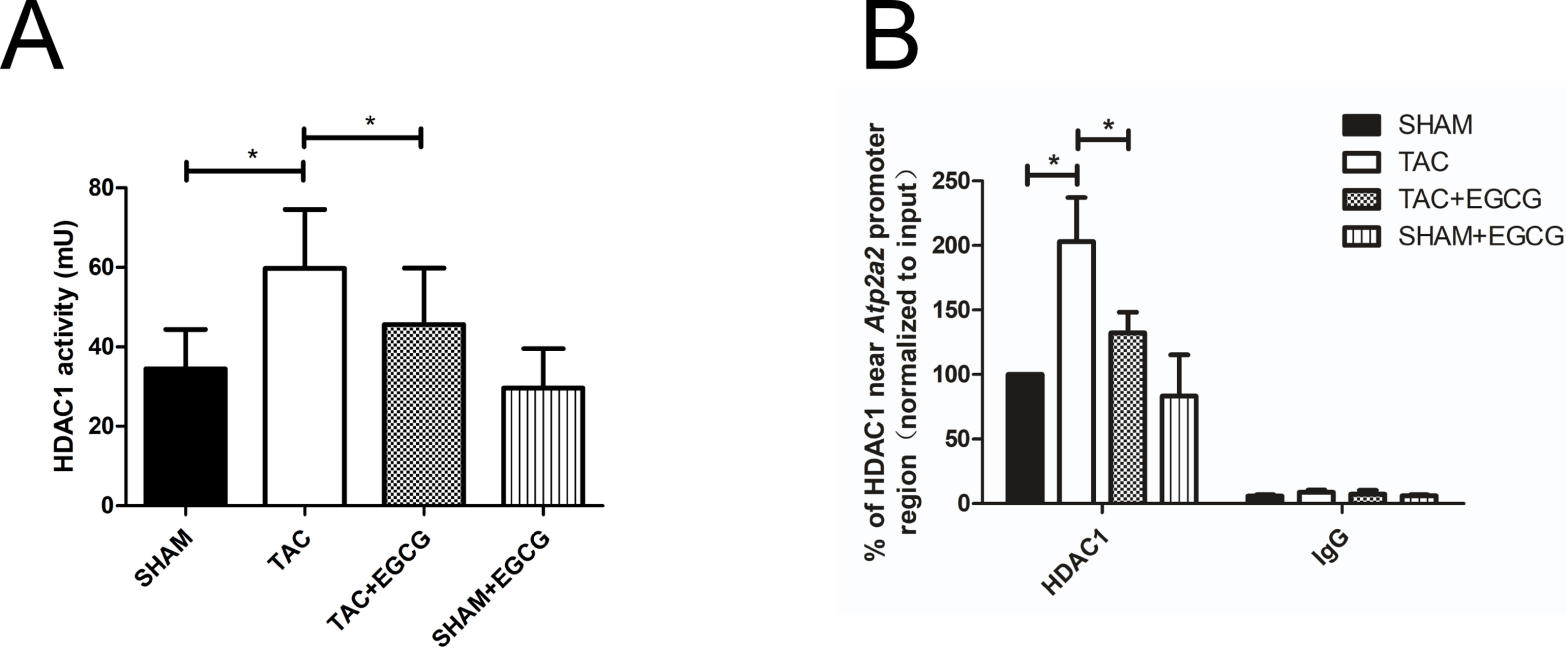
Epigallocatechin-3-gallate (EGCG) inhibits HDAC1 activity and binding near the *Atp2a2* promoter region in mouse cardiac tissue after heart failure. **A**) HDAC1 activity was determined in the hearts of mice with or without EGCG intervention. **B**) Binding levels of HDAC1 near the *Atp2a2* promoter region were higher in the transverse aortic constriction (TAC) group than in the other groups. Values are presented as the mean ± SE. *, *P* < 0.05 by two-way ANOVA; n = 8 per group.

We next determined HDAC1 activity in each group. Fig 6A shows that HDAC1 activity in the TAC groups was enhanced compared to that in the SHAM group, and decreased after EGCG treatment. Thus our data indicates that EGCG can suppress TAC-induced HDAC1 activation. However, this effect was incomplete in TAC mice. EGCG was previously reported to inhibit the binding of HDAC1 to promoters that activate gene expression [26]. Therefore, we detected HDAC1 binding near the promoter region of *Atp2a2* in the four groups. As shown in Fig 6B, these levels were increased in the TAC group, which might correlate with the observed downregulation of AcH3 and AcH3K9. However, binding levels were found to decrease significantly in the TAC+EGCG group.

### 3.6 Binding of transcription factors near the *Atp2a2* promoter

**Fig 7:**
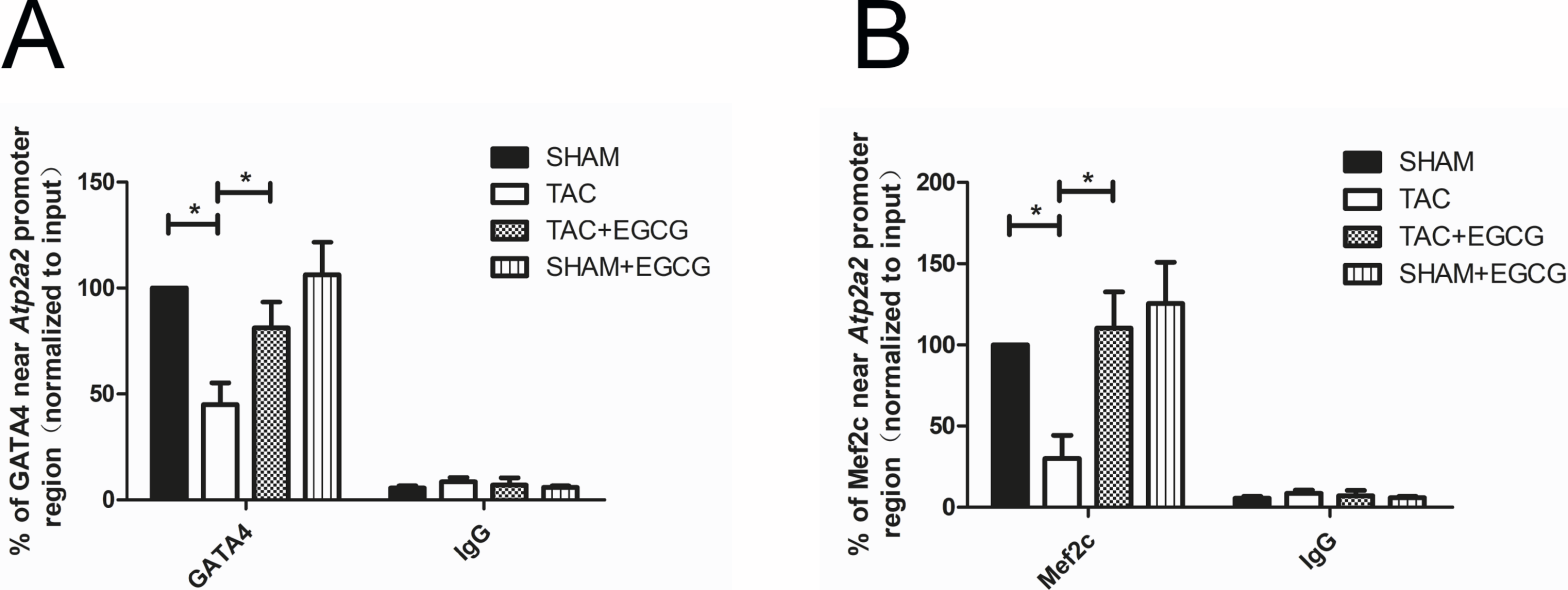
Epigallocatechin-3-gallate (EGCG) upregulates the binding of GATA4 and Mef2c near the *Atp2a2* promoter region in cardiac tissues of mice after heart failure. **A**) Binding levels of GATA4 near the *Atp2a2* promoter region were reduced in hearts of the transverse aortic constriction (TAC) group, which was reversed by EGCG, as assessed by ChIP assays. **B**) Binding levels of Mef2c near the *Atp2a2* promoter region were reduced in hearts of the TAC group, which was reversed by EGCG. Values are presented as the mean ± SE. *, *P* < 0.05 by two-way ANOVA; n = 8 per group.

Cardiac core transcription factors, GATA4 and Mef2c, were shown to play roles in the regulation of *Atp2a2* transcription [27]. Thus, the binding levels of these transcription factors near the *Atp2a2* promoter were determined by ChIP assays. As shown in Fig 7A and 7B, binding levels of GATA4 and Mef2c near the *Atp2a2* promoter region were reduced in heart tissue of the TAC group, which might have been caused by hypoacetylation of histones. This was reversed by the effect of EGCG on the acetylation of histones associated with *Atp2a2*, as the binding of GATA4 and Mef2c was increased significantly after EGCG treatment.

## 4. Discussion

Heart failure continues to be a leading cause of mortality and morbidity worldwide. Conditions that damage or overwork the heart muscle such as hypertension, coronary heart disease, and diabetes can result in heart failure. Hypertension is one of the most important causes of heart failure, as pressure overload exposes cardiac myocytes to elevated mechanical stress and neurohormones, which increases myocardial mass and results in left ventricular hypertrophy. These cardiac changes can further progress to heart failure [28]. Although several types of drugs have been developed over many years, gaps in the treatment of this disease remain. Recently, epigenetic disorders have been reported to play a role in heart failure [18]. Therefore, a better understanding of associated epigenetic mechanisms is required to uncover novel pharmacological agents. Our data indicate that increased HDAC1 activity and binding to the promoter of the gene encoding SERCA2a (*Atp2a2*), which might result in the hypoacetylation of histone 3 lysine 9, in addition to chromatin regulation, result in decreased transcription factor binding to this promoter region. Further investigation showed that EGCG could inhibit the activity and binding of HDAC1 to the *Atp2a2* proximal promoter region to rescue the low expression of SERCA2a and improve cardiac function.

SERCA2a plays a critical role in regulating cardiac function; accordingly, several studies have demonstrated low expression of this protein in heart failure [4–10]. Similar to previous reports, we found that the protein and mRNA levels of *SERCA2a* were decreased after heart failure induced by TAC. However, the underlying epigenetic mechanism of this downregulation of *SERCA2a* remained unclear. Recently, a study demonstrated that histone modification underlies the reprogramming of *SERCA2a* promoters in adult murine left ventricles under conditions of chronic pressure overload [18], indicating a role for the histone-associated epigenetic regulation of *SERCA2a* and epigenetics in the etiology of heart failure. Histone epigenetic modifications include acetylation, methylation, phosphorylation, ubiquitination, and sumoylation [29]. Acetylation is one of the most important histone modifications that can result in chromatin remodeling and the activation of gene expression; moreover, hypoacetylation near the proximal promoter of the gene results in the compaction of chromatin and might affect the binding affinities of transcription factors with the key cis-elements of the proximal promoter [30]. In the present study, our results showed that the binding of total AcH3, as well as the subtype AcH3K9, was reduced, and that the binding of active transcription factors GATA4 and Mef2c was decreased near the *Atp2a2* promoter region after heart failure. However, this pattern was not observed with the AcH3K4 subtype, indicating the selective control of H3 acetylated lysine sites during the regulation of SERCA2a after heart failure. The assessment of other acetylated lysine sites should be performed in future studies.

HDACs are a class of enzymes that remove acetyl groups from ε-N-acetyl lysine amino acids on histones, allowing histones to wrap the DNA more tightly. HDACs can be classified into four classes depending on sequence homology to the original yeast enzymes and domain organization. HDAC1 is one of the most important class I HDACs that regulate histone acetylation; it can enhance electrostatic attraction between DNA and histones and increase chromatin compaction, leading to reduced gene expression. The inhibition of class I HDACs has been reported to suppress pressure overload-induced ventricular hypertrophy and improve systolic function significantly [31, 32], indicating that class I HDACs perform a crucial function during heart failure. Our results showed increased activity and binding of HDAC1 to the *Atp2a2* promoter region, indicating that it might be a causal factor of SERCA2a downregulation mediated by hypoacetylation during TAC-induced heart failure in mice. With respect to the regulation of SERCA2a expression, other subtypes of HDACs should also be assessed in future studies.

Tea polyphenols are the main chemical components of green tea, which contains many types of catechins such as epicatechin, epicatechin-3-gallate, epigallocatechin, and EGCG [33]. EGCG, which has been approved as a health-promoting product for sale, is an ester that forms from the reaction between epigallocatechin and gallic acid, and this compound is associated with important health benefits [34]. EGCG has been reported to exert lipid-lowering effects, in addition to effects on angiogenesis and osteogenesis; it also affects many abnormal pathophysiological changes, as it has been demonstrated to have anti-cancer, anti-inflammatory, anti-collagenase, autoxidative, and anti-fibrotic effects [35–44], and possess therapeutic activity for a number of diseases such as cancer, oral disease, diabetes, and especially cardiovascular diseases [19, 20]. Previously, several mechanisms were suggested for EGCG with respect to heart failure, such as alleviating cardiac fibrosis [45], reducing desensitization of the β1 adrenoceptor [46], modulating the Nrf2/ARE antioxidant system [47], and altering myofilament Ca2+-sensitivity [26, 48]. Recently, studies have demonstrated that EGCG exerts a tremendous effect on events associated with epigenetic regulation, including histone acetylation, methylation, and DNA methylation [22–24]. Pan [26] found that EGCG could inhibit HDAC1 to up-regulate cTnI in aging mice and improve cardiac function, highlighting the detailed mechanisms through which EGCG contributes to epigenetic modifications. Since EGCG is a major epigenetic controller, whether epigenetic regulation by this compound contributes to the prevention of heart failure is not clear. In the present study, we found EGCE could reverse the low expression of SERCA2a observed after TAC; meanwhile, acetylated H3 and H3K9 were upregulated after EGCG treatment. We further found that HDAC1 activity and binding were decreased with EGCG treatment. All of these findings indicate that EGCG could inhibit HDAC1 activity and binding to the promoter of the gene encoding SERCA2a, resulting in increased expression through the enhanced binding of AcH3 and AcH3K9 to the proximal promoter of this gene.

In conclusion, upregulation of SERCA2a via the modification of histone acetylation plays a role in the EGCG-mediated prevention of pressure overload-induced heart failure, and thus might represent a novel pharmacological agent for the treatment of heart failure.

## Supporting information

S1 Text Protocol for TAC. Detailed procedure of Transverse Aortic Constriction (TAC).
(DOCX)

S2 Text Protocol for Echocardiography. Detailed procedure of echocardiography in mice.
(DOCX)

S1 Table The diameter and blood flow velocity of the transverse aorta. The transverse aorta diameter decreased significantly after the TAC operation. The velocity of the transverse aorta blood flow increased significantly after TAC.
(XLSX)

S2 Table Echocardiographic parameters before and 12 weeks after operation. Compared to that before the operation, the thickness of the left ventricular anterior wall (LVAW) and left ventricular posterior wall (LVPW) increased in TAC group hearts. The left ventricular chamber was dilated, and the ejection fraction (EF) and fraction shortening (FS) were decreased in the TAC group but not in TAC+EGCG group hearts.
(XLSX)
