# Supplementary material for: Epigallocatechin-3 gallate prevents pressure overload-induced heart failure by up-regulating SERCA2a via histone acetylation modification in mice
Source: PLoS One. 2018 Oct 4;13(10):e0205123. doi: 10.1371/journal.pone.0205123 (PMC6171916; doi:10.1371/journal.pone.0205123)
Supplement: S1 Text — Detailed procedure of Transverse Aortic Constriction (TAC). (DOCX) [file pone.0205123.s001.docx]

1. Isofluorane was given by inhalation via a specially designed nose cone at 3% in air delivered at 100-200ml/min for induction and 1.5-2.5% in air at 100-200ml/min for maintenance.

2. Mice were placed supine on a heating pad under a lamp (to maintain body temperature at 36-37◦C) and operating microscope.

3. Fur was removed from the neck and upper chest area, and the area was wiped with 75% ethanol.

4. A small horizontal skin incision 7-10 mm in length was made at the level of the suprasternal notch.

5. Once the trachea was located, a 5 mm longitudinal cut was made down the sternum, the thymus was retracted and the aortic arch located.

6. A curved 27-gauge needle was used to place a 6–0 silk suture under the aorta arch between the origin of the right innominate and left common carotid arteries. A 27-gauge needle was used to control the tightness of the suture.

7. After ligation of the arch, skin was closed with 4-0 Nylon suture and the mouse was allowed to recover in a clean cage on a heating pad until fully recovered.

8. Buprenorphine was administered subcutaneously once per day for 3 days for post-operative analgesia.
