# Supplementary material for: Epigallocatechin-3 gallate prevents pressure overload-induced heart failure by up-regulating SERCA2a via histone acetylation modification in mice
Source: PLoS One. 2018 Oct 4;13(10):e0205123. doi: 10.1371/journal.pone.0205123 (PMC6171916; doi:10.1371/journal.pone.0205123)
Supplement: S2 Text — Detailed procedure of echocardiography in mice. (DOCX) [file pone.0205123.s002.docx]

**PREPARATION**

1. Anesthetize in a Perspex chamber with 3% isofluorane in oxygen for induction, and 1-1.5% in oxygen at 1 l/min for maintenance.

2. Eye lubricant should be placed on each eye to prevent drying of the area.

3. Place the mouse on a heating pad under a lamp, a rectal probe should be inserted to monitor the temperature of the animal during the imaging session, maintain body temperature at 36.5-37.5 °C.

4. A small amount of ECG gel should be placed on the copper leads on the platforms and the paws taped to them.

5. Hair on the precordial region was removed with depilatory cream.

**ECHO EXAM**

**To turn system ON:**1. On the rear panel, push up the **Main Power** switch. This connects the system to the power source and turns on the internal fans, but it does not turn on the control.
2. On the left side of the control panel module, press the **Computer Standby** switch. This is a toggle switch, so when you press it, it does not stay pushed in like a light switch. Instead it returns to its original position. This is normal. The system starts the control panel backlights, the display monitor and the computer operating system.

**B-Mode**B-Mode is the imaging mode you will work with most often because it is the most effective mode for locating anatomical structures. If you have seen a conventional ultrasound image then you are already familiar with B-Mode.

*To acquire a B-Mode image*

**1.** Press **B-Mode**
The **B-Mode** imaging window appears and the system begins storing cine loop data in the acquisition buffer.
**2.** Position the transducer and locate your region of interest.
**3.** If the image orientation looks backward to you, click the image orientation icon or (on the control panel press **invert**) to flip the image view horizontally

**4.** Adjust the **image width** control to remove image content outside the region of interest to optimize the image data for analysis.
**5.** Press **presets** to cycle through the available presets and then select an appropriate set of optimized image acquisition settings.
**6.** On the control panel, adjust the B-Mode controls (page 194) to refine your image acquisition settings if required.
**7.** Press the **scan/freeze** toggle control to stop the data acquisition so you can review the data in the acquisition buffer.
**8.** Roll the trackball side to side to scroll through the cine loop.
**9.** If you are satisfied with the cine loop or an individual image frame, store your image data.
 To save a cine loop press **cine store**.
 To save and label a cine loop, press **image label**.
 To save the displayed image frame press **frame store**.
**10.** Press **scan/freeze** toggle control to resume scanning.
**11.** Save images as required.
**12.** Press **close**. The system closes the series you are working on and displays the **Study Information** window.
**13.** Complete the required fields to define your study and click **OK**.

**M-Mode**M-Mode is used primarily to measure the movement and dimensions of cardiac structures such as chambers and walls.

*To acquire an M-Mode image*

**1.** Start imaging in B-Mode and position the transducer to situate your region of interest in the center of the image area.
**2.** Adjust the **image width** control to remove image content outside the region of interest to optimize the image data for analysis.
**3.** Press **M-Mode**.
The system begins acquiring B-Mode image data and displays the yellow M-Mode sample gate overlay on the B-Mode image.

**4.** Press **update** or press **M-Mode** again.
The dual-window **M-Mode** image area workspace appears. The M-Mode window is on the bottom, the B-Mode scout window is on the top. The system begins storing cine loop data in the acquisition buffer, and live acquisition data appears in both windows.
**5.** (Optional) To display a larger B-Mode window so you can guide the position of your transducer more precisely:
a. Press **update** to display the full B-Mode window.
b. When you have positioned your transducer, press **update** again to return to the dual-window workspace.
**6.** Press **presets** to cycle through the available presets and then select an appropriate set of optimized image acquisition settings.
**7.** On the control panel, adjust the M-Mode controls (page 229) to refine your image acquisition settings if required.
**8.** Press the **scan/freeze** toggle control to stop the data acquisition so you can review the data in the acquisition buffer.
**9.** Roll the trackball side to side to scroll through the cine loop.
**10.** If you are satisfied with the cine loop or an individual image frame, store your image data.
 To save a cine loop press **cine store**.
 To save and label a cine loop, press I **image label**
**11.** Press **scan/freeze** toggle control to resume scanning.
**12.** Save images as required.
**13.** Press **close**. The system closes the series you are working on and displays the **Study Information** window.
**14.** Complete the required fields to define your study and click **OK**.

**PW (Pulsed Wave) Doppler Mode**PW Doppler Mode (Pulsed Wave Doppler) is an ultrasound mode you can use to measure the velocity and direction of flow. The Vevo software presents the detected PW Doppler signal as both a spectral image in the display window as well as an audio output through the system speakers.

*Acquiring PW Doppler Mode images*

**1.** In B-Mode, position the transducer to situate your region of interest in the center of the image area.
**2.** Set the PW Doppler sample volume (page 257)

**3.** Adjust the **image width** control to remove image content outside the region of interest to optimize the image data for analysis.
**4.** Press **PW**.
The system displays the yellow sample volume overlay on the B-Mode image.
**5.** Press **PW** again.
The dual-window **PW Doppler Mode** workspace appears. The PW Doppler Mode window is on the bottom, the B-Mode scout window is on the top.
**6.** The system begins storing cine loop data in the acquisition buffer.
**7.** Press **presets** to cycle through the available presets and then select an appropriate set of optimized image acquisition settings.
**8.** On the control panel, adjust the PW Doppler Mode controls (page 251) to refine your image acquisition settings if required.
**9.** Press the **scan/freeze** toggle control to stop the data acquisition so you can review the data in the acquisition buffer.
**10.** Roll the trackball side to side to scroll through the cine loop.
**11.** If you are satisfied with the cine loop, store your image data.
 To save a cine loop press **cine store**.
 To name the image you just stored, press **image label**.
**12.** Press S **scan/freeze** toggle control to resume scanning.
**13.** Save images as required.
**14.** Press **close**. The system closes the series you are working on and displays the **Study Information** window.
**15.** Complete the required fields to define your study and click **OK**

**Color Doppler Mode**Color Doppler Mode uses Doppler principles to determine the mean velocities of blood within the region of interest. The system then applies *color* that represents these various velocities under the convention of BART (Blue=Away Red=Toward).

*To acquire a Color Doppler Mode image*

**1.** Press **color**. In the image area:
 The system begins storing cine loop data in the acquisition buffer
 The system displays the region-of-interest (ROI) box overlay on the BMode background image. If your transducer is positioned almost parallel over a vessel, the system displays color data in the ROI box
**2.** To change the size and proportion of the color ROI box:
a. Press **update**. The color ROI box becomes a dashed-line box.

b. Trackball up or down to change the height of the box, or left and right to
change the width of the box.
c. Press U **update** e to return to the solid-lined color ROI box.
**3.** To change the position of the box, trackball to move the color ROI box.
**4.** Press P **presets**  to cycle through the available presets and then select an appropriate set of optimized image acquisition settings.
**5.** On the control panel, adjust the Color Doppler Mode controls (page 309) to refine your image acquisition settings if required.
**6.** Press the **scan/freeze** toggle control to stop the data acquisition so you can review the data in the acquisition buffer.
**7.** Roll the trackball side to side to scroll through the cine loop.
**8.** If you are satisfied with the cine loop or an individual image frame, store your image data.
 To save a cine loop press C **cine store** .
 To save and label a cine loop, press I **image label**.
 To save the displayed image frame press F **frame store**.
**9.** Press **scan/freeze** toggle control to resume scanning.
**10.** Save images as required.
**11.** Press **close**. The system closes the series you are working on and displays the
**Study Information** window.
**12.** Complete the required fields to define your study and click **OK**.

**Imaging Guide Outline**

*I. Parasternal Long Axis View (PLAX)*

Scanhead position: Place the transducer in vertical fashion, with the notch pointing towards the animal’s head. Then rotate the transducer approximately 35° counter-clockwise.

*II. Parasternal Short Axis View (PSAX)*

Scanhead position: From the PLAX view, parasternal short axis view can be obtained by rotating the scanhead 90° clockwise. Following this 90° rotation, the Y-axis may need further adjustment to obtain the proper view.

*III. Apical Four Chamber View*

Scanhead position: From the short axis view, dramatically angle the animal’s head down and also angle the base of the probe toward you. This is essentially to achieve a coronal view of the heart looking up towards the apex.

*IV. Aortic Arch View*

Scanhead position: The aortic arch view is obtained from a modified right parasternal view. The scanhead is positioned with the notch pointing towards the chin of the animal. The scanhead may need to be rotated slightly clockwise to optimize the image.

**To turn your system OFF:
1.** Ensure that you have stored all the image data that you are working on.
**2.** Press the **Computer Standby** switch.
The computer shuts down, the monitor powers down, and the control panel
backlights turn off. The fans continue to run.
**3.** If you need to turn off all power to the system:
a. Let the fans run for 10 minutes to safely cool down the internal
components.
b. Push down the **Main Power** switch

**End**

The mouse was allowed to recover in a clean cage on a heating pad until fully recovered.
